# Pancreatic Islets Accumulate Cadmium in a Rodent Model of Cadmium-Induced Hyperglycemia

**DOI:** 10.3390/ijms22010360

**Published:** 2020-12-31

**Authors:** Ryan Fitzgerald, Andrew Olsen, Jessica Nguyen, Winifred Wong, Malek El Muayed, Joshua Edwards

**Affiliations:** 1 Chicago College of Osteopathic Medicine, Midwestern University, Downers Grove, IL 60515, USA; rsfitz6@gmail.com (R.F.); a.b.olsen@gmail.com (A.O.); 2 Chicago College of Pharmacy, Midwestern University, Downers Grove, IL 60515, USA; j10nnguyen@gmail.com; 3 Division of Endocrinology, Metabolism, and Molecular Medicine, Feinberg School of Medicine, Northwestern University, Evanston, IL 60208, USA; winifred.wong@northwestern.edu (W.W.); m-muayed@northwestern.edu (M.E.M.); 4 College of Graduate Studies, Midwestern University, Downers Grove, IL 60515, USA

**Keywords:** cadmium, pancreatic islets, insulin, zinc, blood glucose, type II diabetes mellitus

## Abstract

Cadmium (Cd) is an anthropogenic as well as a naturally occurring toxicant associated with prediabetes and T2DM in humans and experimental models of Cd exposure. However, relatively few studies have examined the mechanism(s) of Cd-induced hyperglycemia. The purpose of this study was to examine the role of pancreatic islets in Cd-induced hyperglycemia. Male Sprague–Dawley rats were given daily subcutaneous doses of Cd at 0.6 mg/kg over 12 weeks. There was a resulting time-dependent increase in fasting blood glucose and altered insulin release in vitro. Islets isolated from control (saline-treated) and Cd-treated animals were incubated in low (0.5 mg/mL) or high (3 mg/mL) glucose conditions. Islets from 12 week Cd-treated animals had significantly less glucose-stimulated insulin release compared to islets from saline-treated control animals. The actual Cd content of isolated islets was 5 fold higher than the whole pancreas (endocrine + exocrine) and roughly 70% of that present in the renal cortex. Interestingly, islets isolated from Cd-treated animals and incubated in high glucose conditions contained significantly less Cd and zinc than those incubated in low glucose. These results show that within whole pancreatic tissue, Cd selectively accumulates in pancreatic islets and causes altered islet function that likely contributes to dysglycemia.

## 1. Introduction

Cadmium (Cd) is a common industrial and naturally occurring environmental toxicant that is carcinogenic with toxic effects at multiple sites including; lung, liver, testicular, kidney and bone [1]. Cigarette smoke is the main source of non-occupation exposure to Cd. While the kidney is considered the primary target organ of Cd toxicity with tissue concentrations being the highest in the renal cortex at concentrations up to 20 fold higher than that of other tissues with the order of accumulation being: kidney cortex >> liver > thyroid > pancreas > bone [2,3,4]. The biological half-life of Cd is 10–30 years and individuals with low-level, non-occupational exposure to Cd reach peak body burden at age 50 [5]. The mechanism responsible for the long half-life is unknown. However, it is believed that Cd-conjugates within cells undergo lysosomal degradation during which Cd becomes uncoupled, then it is transported to the cytoplasm to become re-conjugated with metallothionein, for review see [6].

While the nephrotoxic effects of Cd are well known reports show significant correlations between exposure to Cd and the prevalence of prediabetes and/or type II diabetes mellitus (T2DM) in countries such as Pakistan [7,8], China [9], USA [10,11], and Australia [12]. Other studies do not show a statistically significant correlation between Cd exposure and diabetes [13,14]. However, a concern of the Menke et al., 2016 report was that urinary Cd, as a proxy of Cd exposure, is a confounding factor when examining data from patients with frank diabetes mellitus [15].

Short-term and long-term experimental models of exposure have shown Cd to cause hyperglycemia and disrupt glucose homeostasis [2,16,17,18,19,20]. In ovariectomized cynomolgus monkeys given various doses of Cd over 13–15 months, animals in the highest Cd dosing group had increased fasting blood glucose, decreased fasting blood insulin and islets of Langerhans that were atrophic with vacuolization and decreased insulin labelling [21]. The mechanism of action by which Cd causes disruption of glucose homeostasis is unknown. However, insulin-secreting pancreatic β cell dysfunction is a possible factor. It has previously been shown that Cd avidly accumulates in insulin-producing β-cells in vitro, resulting in impaired insulin secretion [22]. For individuals with elevated levels of Cd exposure due to occupation, fasting blood insulin levels were significantly lower [23]. Additional mechanisms by which Cd may alter blood glucose levels involve changes in glucose transporters in adipocytes [18] or increase gluconeogenesis in the kidney and/or liver [24,25], thereby contributing towards insulin resistance. Taken together, this would indicate that the diabetogenic effects of Cd are multifaceted and complex.

The objective of the current study was to examine and identify the diabetogenic effects of Cd using a well-characterized animal model of Cd-induced nephrotoxicity. This model has previously been used by the co-author (Edwards) to report on Cd-induced elevation in blood glucose [16,26]. Specifically, pancreatic islet function was examined as well as the ability of islets to accumulate Cd during in vivo exposure. Islet zinc (Zn) content was also examined because Cd could potentially use the same transmembrane transporters as well as binding proteins to gain access to intracellular compartments in pancreatic β-cells [22].

## 2. Results

In general, the animals tolerated the Cd exposure very well. However, the Cd treated animals gained significantly less body weight after 11 weeks of exposure with differences in average bodyweight beginning to appear at week 4 [27] and injection-site subcutaneous tumors appeared after 5–6 weeks of treatment as reported in [28]. Furthermore, renal injury as determined by increases in the appearance of proteinuria and polyuria were evident at week 11 as reported in [16,29] in which the same animal model of chronic Cd intoxication was used.

### 2.1. Long Term Cd Exposure Resulted in Time-Dependent Increase in Fasting Blood Glucose

**Figure 1:**
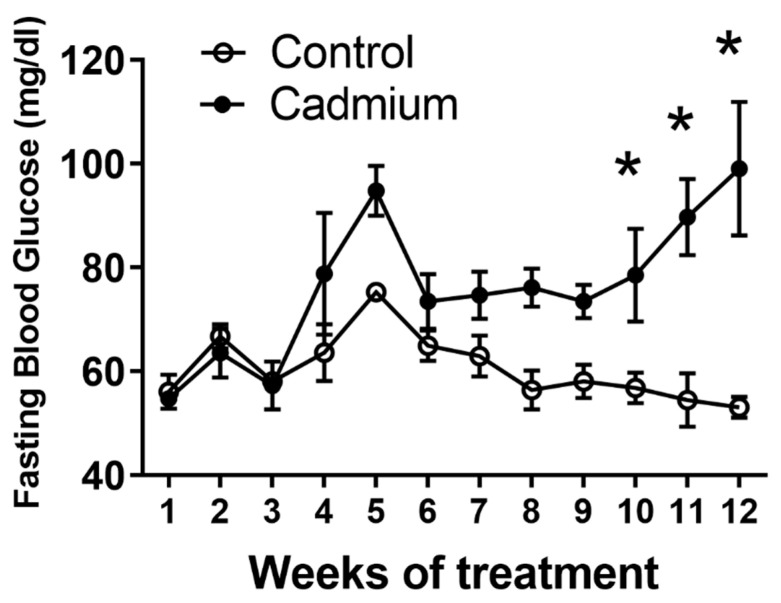
Animals were treated with Cd (0.6 mg/kg/day, five days a week for 12 weeks). Weekly, 24 h fasting blood glucose values were measured using an Equaline brand blood glucose meter with disposable test strips. Values are mean ± SE. An asterisk (*) denotes significant differences from week 1 values (one-way ANOVA and Tukey’s post tests, *p* ≤ 0.05, *n* = 3–10 for each data point).

Blood glucose levels increased over time during the study in 24 h fasted animals, see Figure 1. At 12 weeks of treatment, the mean fasting blood glucose for control animals was 53 ± 2.0 mg/dL and 99 ± 16.4 mg/dL (mean ± SE) for Cd-treated animals. The relatively long 24 h time point was chosen because these animals were also part of another study examining Cd-induced nephrotoxicity and animals were fasted for 24 h for urine collection to test renal function.

### 2.2. Fasting Serum Insulin is Reduced During Cd Exposure

**Figure 2:**
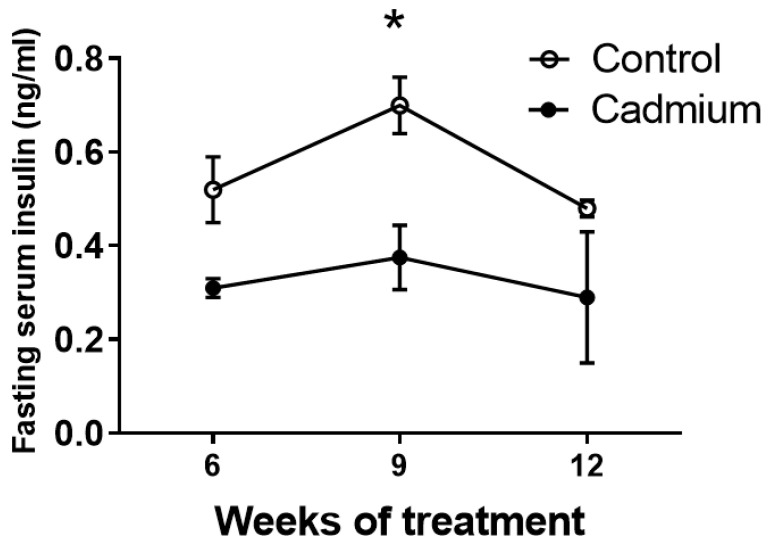
Serum samples were taken from 5 h fasted animals at 6, 9, and 12 weeks of Cd treatment (0.6 mg/kg/day). An asterisk (*) denotes significant differences from week-matched control values (one-way ANOVA and Tukey’s post tests, *p* ≤ 0.05, *n* = 3 for each data point).

Serum insulin levels were measured from 5 h fasted animals with mean values decreased at weeks 6, 9 and 12, see Figure 2. However, a statistically significant difference between the saline- and Cd-treatment groups was evident only at the week 9 time point.

### 2.3. Islets From Cd-Treated Rats Have Altered Insulin Release

**Figure 3:**
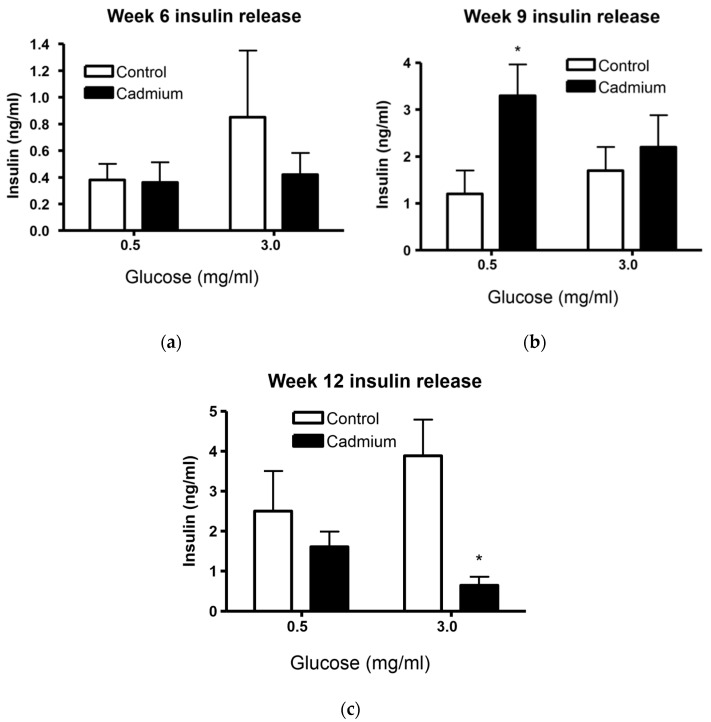
Data show changes in insulin release in freshly isolated islets from week-matched control animals and Cd-treated (0.6 mg/kg/day) animals after 6 (**a**), 9 (**b**), and 12 weeks (**c**). An asterisk (*) denotes significant treatment differences from islets incubated in the same glucose concentrations 0.5 mg/mL (week 9) or 3.0 mg/mL (week 12) (one-way ANOVA and Tukey’s post tests, *p* ≤ 0.05, *n* = 3–8 for each data point).

Figure 3 shows the long-term effects of Cd exposure on basal or low glucose (0.5 mg/mL) as well as glucose-stimulated or high glucose (3.0 mg/mL) insulin release. No changes in basal or glucose-stimulated insulin release was detected at week 6, see Figure 3a. However, insulin release from islets incubated in basal glucose (0.5 mg/mL) was significantly higher from week 9 Cd-treated animals as compared to saline-control treated animals (Figure 3b). There was a paradoxical reversal in the trend of increased insulin release from islets in week 9 Cd-treated animals compared to week 12 treated animals (Figure 3c). There were no significant changes in basal or glucose-stimulated insulin release when individual islet data were transformed to represent percent insulin release (insulin release/total islet insulin multiplied by 100).

### 2.4. Total Insulin Content from Islets Isolated from Week 9 Cd-Treated Animals Was Significantly Increased

**Figure 4:**
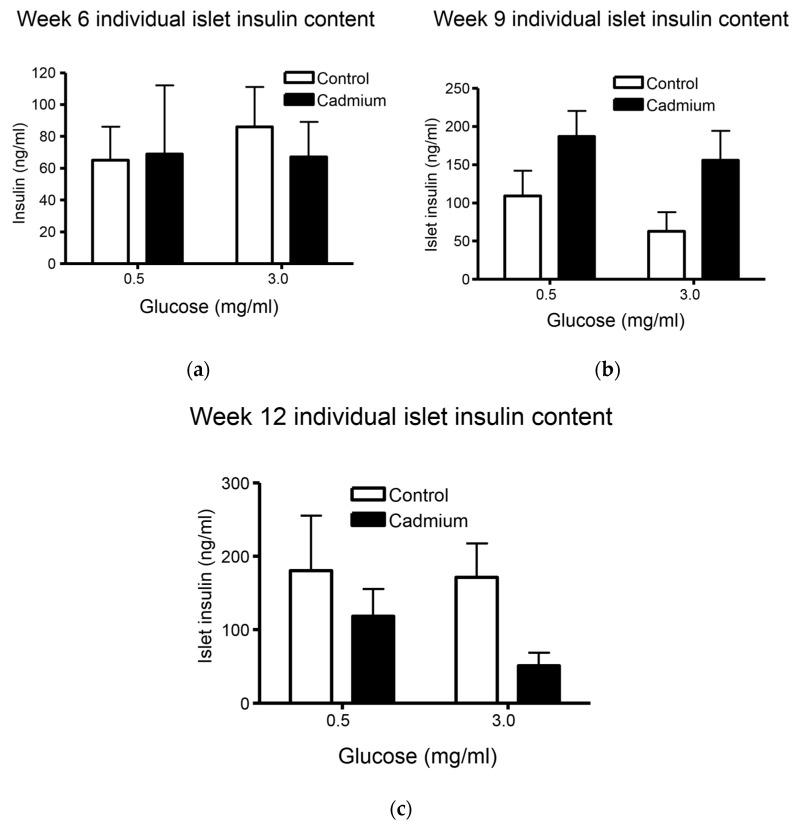
Data show changes in insulin content in freshly isolated islets from week-matched control animals and Cd-treated (0.6 mg/kg/day) for 6 (**a**), 9 (**b**), and 12 weeks (**c**). Two-way ANOVA showed significant differences when comparing islet insulin content from control vs. Cd-treated animals (*p* = 0.0139) after nine weeks of treatment. However, no significant differences were detected between week-matched control vs. Cd-treated animals following post-hoc Tukey’s multiple comparison test (*p* > 0.05 for all week matched comparisons); *n* = 3–8 for each data.

We found a lack of effect of long term Cd exposure on islet insulin content, see Figure 4. Others have shown that Cd causes decreased insulin mRNA expression; however, no change in actual insulin content was reported [2]. We did not find any statistically significant changes in insulin content from freshly isolated islets when performing the post-hoc Tukey’s test. However, two-way ANOVA did show a significant increase in islet insulin content from islets isolated from week 9 Cd-treated animals when 0.5 and 3.0 mg/mL glucose treatment groups were combined (*p* = 0.0137). Similar to data shown in Figure 3c above, there was a near statistically significant (*p* = 0.07) decrease in islet insulin content from 12 week Cd-treated animals when 0.5 and 3.0 mg/mL glucose treatment groups were combined. There were no changes in insulin content at the earliest 6 week time point, see Figure 4a.

### 2.5. Cadmium Selectively Accumulates within the Islets of Langerhans within Whole Pancreatic Tissue

**Figure 5:**
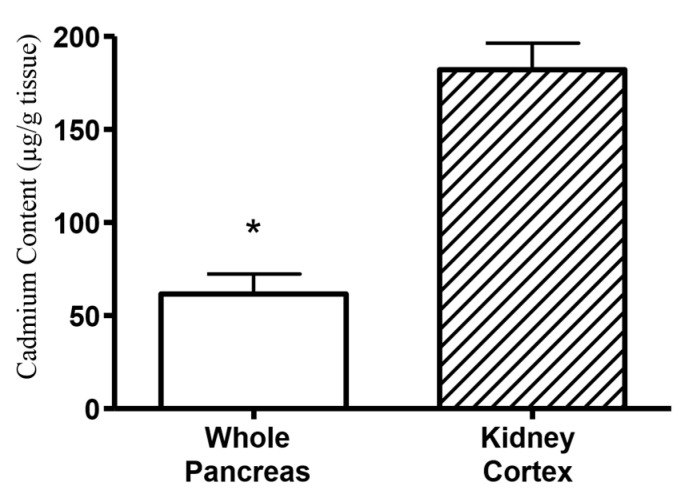
Data represent Cd content of whole pancreas and kidney cortex from 12 week Cd treated (0.6 mg/kg/day) animals. An asterisk (*) denotes significant differences (*t*-test, *p* ≤ 0.05, *n* = 4 for each data point).

We found that Cd content in the whole pancreas (endocrine and exocrine) is much less compared to the renal cortex following 12 weeks of Cd treatment, see Figure 5.

**Figure 6:**
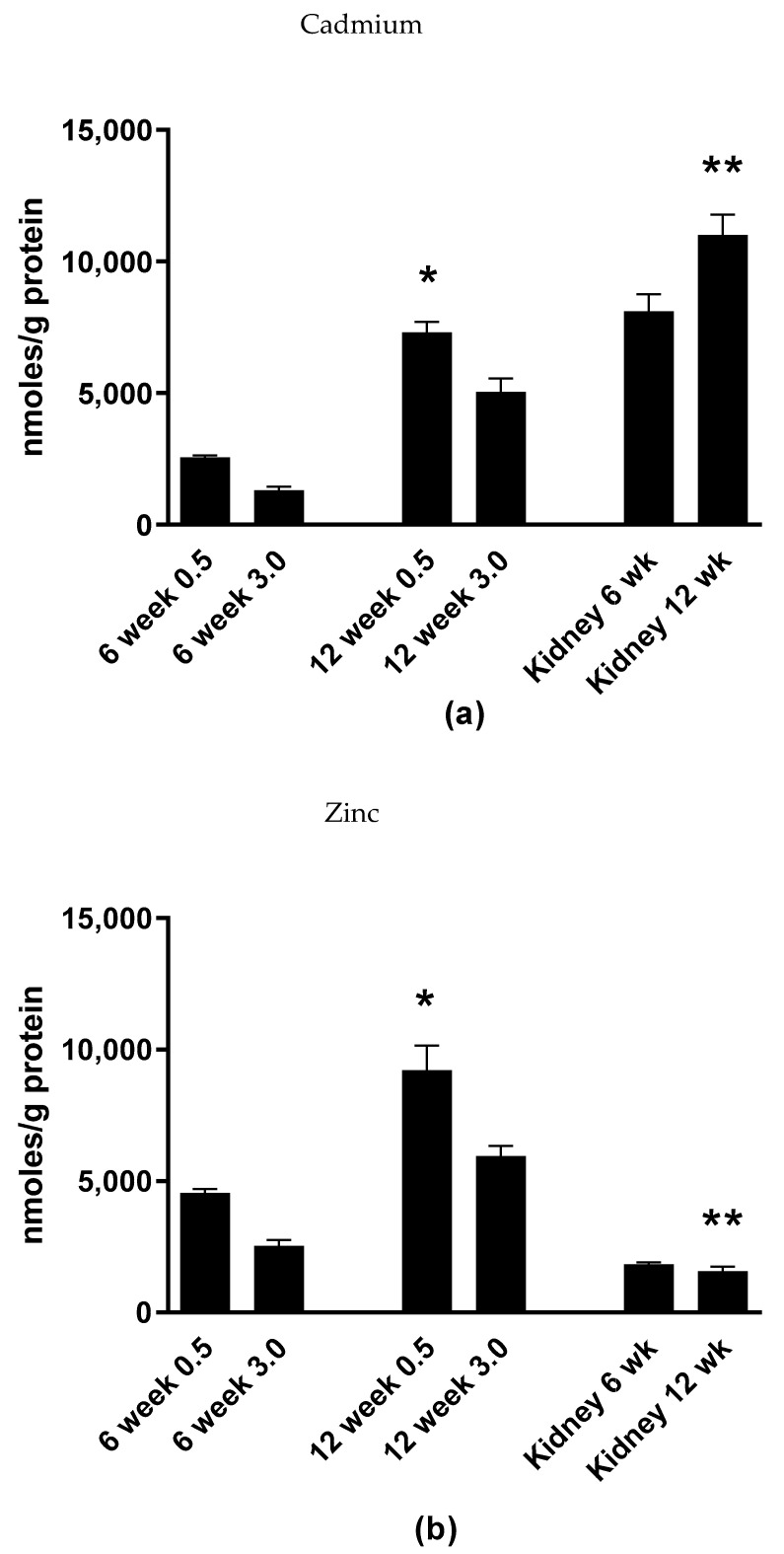
Data show Cd (**a**) or Zn (**b**) accumulation within isolated islets and renal cortex from animals treated with Cd (0.6 mg/kg/day) for 6 or 12 weeks. Islets were incubated in either low “0.5” or high “3.0” glucose; values are glucose concentrations in mg/mL. For both Cd (**a**) and Zn (**b**) asterisk (*) indicates statistically significant differences as compared to the 12 week 3.0 mg/mL glucose islet treatment group (ANOVA followed by Tukey’s multiple comparison test, *p* ≤ 0.05). Double asterisks (**) indicate significant differences as compared to islet lysates isolated from 12 week Cd treated animals and incubated in 0.5 mg/mL glucose; *n* ≥ 3 for each treatment group. For zinc (**b**) data set only, other statistically significant differences detected were between treatment groups were: 12 week (0.5) control vs. 12 week (0.5) Cd treated; six week (0.5) Cd treated vs. 12 week (0.5) Cd treated and six week (3.0) Cd treated vs. 12 week (3.0) Cd treated. *n* ≥ 3 for each treatment group.

These data show the typical distribution of Cd reported in the literature with the whole pancreas containing roughly 1/5th of that of the renal cortex [2]. Figure 6a shows the Cd content of acutely dissociated islets isolated from animals treated after 6 and 12 weeks of Cd treatment. Compared to the rental cortex, labeled “kidney” on the X axis, there was less Cd in the islets. However, the difference in Cd accumulation was only approximately 70% less in islets incubated in low glucose (0.5 mg/mL) as compared to the renal cortex in animals treated with Cd for 12 weeks. Of interest is the effect of high vs. low glucose on islet Cd and Zn content. At 12 weeks of treatment, there was a significant decrease in islet Cd content when islets were incubated in high glucose and underwent glucose-stimulated insulin release (Figure 6a).

The same situation occurs with Zn whereas islet Zn content is decreased when islets are incubated in high glucose compared to low glucose conditions, see Figure 6b. Islets from saline control animals were not examined for Cd or Zn levels. These results indicate that Cd in islets is labile and has a dynamic relationship with islet cells, presumably β-cells, involved in glucose-stimulated insulin secretion.

### 2.6. Cadmium Alters the Histopathology and Morphology of Islets of Langerhans

Table 1 shows a semi-quantitative analysis of histopathological markers of islets of Langerhans in pancreatic tissue as determined by a trained veterinary pathologist. Of all the factors quantified, vacuolation was the most altered with Cd treatment.

Morphometric analyses were also performed on H&E-stained or glucagon- or insulin-immunolabeled pancreatic tissue. Islets in H&E stained tissue sections were quantified for: mean diameter, minimum diameter, maximum diameter, roundness, surface area, perimeter, number of nuclei per surface area, ratio of insulin to glucagon positive cells, number of insulin per surface area and number of glucagon per islet surface area, see Table 2. Number of cells normalized to islet surface area were significantly lower in the Cd-treatment group at all time points examined: weeks 6, 9, and 12. Minimum diameter was also significantly different however, this was due to an unexplained decrease in the value for control-treated animals at week 9.

Furthermore, pancreatic tissue immuno-labeled for both insulin and glucagon did not show significant differences regarding: number of insulin/glucagon immune-positive cell ratio, number of insulin positive cells normalized to islet surface area, number of glucagon positive cell normalized to surface area. However, the data set for insulin/glucagon ratio approached statistical significance (*p* = 0.088), see Table 3.

**Figure 7:**
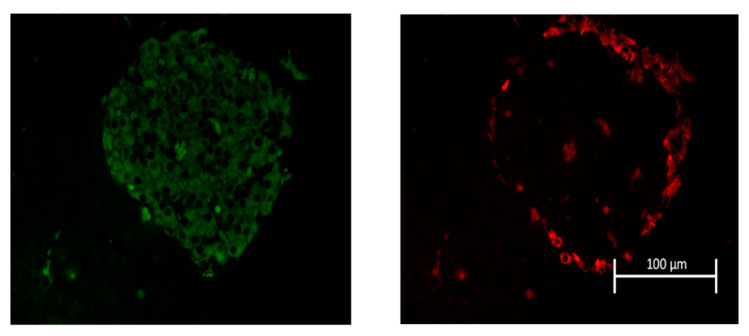
Representative image of insulin and glucagon labelled islet of Langerhans. Pancreatic tissue was collected from 6, 9 and 12 week control- and Cd-treated rats (0.6 mg/kg/day, five days per week, for Cd and equal volumes of saline for control s.c.). Tissue was then immuno-labelled for the presence of β-cells with insulin (green) left panel and for α-cells with glucagon (red) right panel.

The representative images in Figure 7 show insulin and glucagon labeling in pancreatic tissue from a control-treated animal. In all experiments, the localization of glucagon-positive α-cells (red) occurs at the periphery of the islets in rats, whereas α-cells are more evenly dispersed throughout islets in humans and mice.

### 2.7. Pancreatic Islet Density Was Less in Cd-Treated Animals

**Figure 8:**
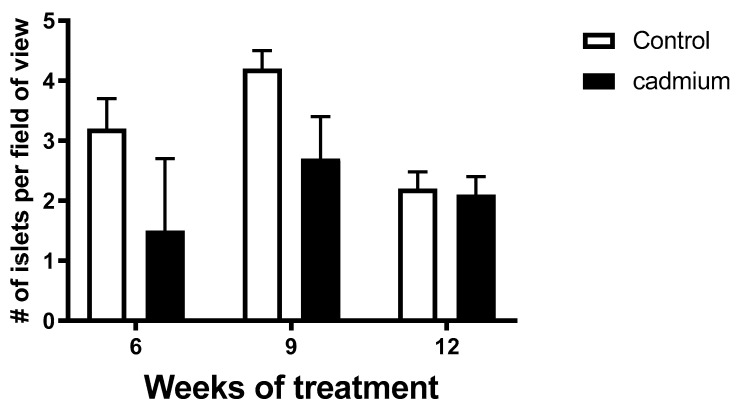
Pancreatic islet density was quantified from H&E stained tissue collected from control- and Cd-treated animals after 6, 9, and 12 weeks of dosing (0.6 mg/kg/day, five days per week, for Cd and equal volumes of saline for control s.c.). 2-way ANOVA showed significant differences when comparing islet density from control vs. Cd-treated animals (*p* = 0.0154). No significant differences were found following post-hoc Tukey’s multiple comparison test (*p* > 0.05 for all week matched comparisons). Two to three images were captured per tissue section, islet number quantified and averaged; *n* = 3–6 for each treatment group at each time point.

Figure 8 shows pancreatic islet density data from 6, 9, and 12 week control- and Cd-treated animals. While 2-way ANOVA showed significant differences (*p* = 0.0154) in islet density when all data from control and Cd-treated tissues were analyzed, no significant differences were detected for week-matched control vs. Cd treatment groups (*p* > 0.05). Also of note is that the longest time point examined at 12 weeks, shows the least differences in average islet density values for control vs. Cd treatment groups.

## 3. Discussion

The findings presented here reinforce the diabetogenic effects of the environmental contaminant Cd and for the first time show that Cd selectively accumulates within islets of Langerhans at levels similar to that of the renal cortex in an animal model of Cd exposure. Furthermore, the amount of Cd present in isolated islets was significantly decreased when the islets were incubated in high glucose conditions and underwent glucose stimulated insulin release. This may contradict prior data that show a long half-life of more than 20 years that was previously implicated for other tissue. If confirmed with further data, this data may hold hope for a gradual reduction in islet Cd content in the event of successful interventions aimed at decreasing exposure levels. Zinc islet content displayed the same pattern of decreased levels when islets were incubated in high glucose conditions. This may point to overlapping transport and buffering pathway. It has been well described that several zinc transporters of the ZIP family as well as the main intracellular Zn binding proteins, such as metallothionein transport/bind protein, in similar quantities [30,31,32,33,34,35]. Semi-quantitative histopathological scoring suggested elevated vacuolation, apoptosis, pyknosis and dissociation. However, only islet cell density was decreased with no other changes found in islet morphology, the number or density of insulin- or glucagon-immunopositive β- or α-cells, respectively.

The Edwards lab has previously shown a gradual elevation in fasting blood glucose in the same subchronic model of Cd toxicity reported here [16]. Figure 1 of the current study shows a more complete time course response with statistically significant increases occurring one week earlier at 10 weeks of Cd treatment compared to the previous report [16]. Figure 2 shows a significant decrease in fasting serum insulin after nine weeks of treatment. However, it should be noted that other reports from the Edwards lab using different methodologies to determine serum insulin in the same animal model of chronic Cd toxicity did not show significant changes in serum insulin [26]. Subtle differences in the collection and processing of serum samples could potentially account for these differences.

The most profound finding of the current study is that Cd selectively accumulates within pancreatic islets following in vivo dosing (Figure 5 and Figure 6a). Furthermore, Cd appears to exit the islet in the presence of elevated glucose. One serendipitous experimental detail in the current study is that only 90 min of time elapsed between the time the islets completed the isolation process and then incubated in either low or high glucose. Normally, isolated islets are maintained in culture overnight to allow for recovery from the enzymatic digestion. Figure 3 shows that the average value of insulin release is higher in the high glucose compared to low glucose treatment group in islets isolated from saline-control treated animals although not statistically significant. However, the 90 min recovery time following the isolation process makes the finding that Cd (Figure 6a) was significantly decreased in high as compared to low glucose conditions more compelling. This indicates that the outflow of islet Cd may be very sensitive to β-cell depolarization or excitability but occurs independent of insulin exocytosis. It should be noted that the glucose concentration of the recovery cell culture media that the islets were incubated in contained 2 mg/mL glucose. This approaches the 3 mg/mL of glucose in the high glucose buffer. Therefore, it is likely that a significant portion of Cd may have undergone exocytosis prior to the glucose challenge and as a result the actual Cd concentration would be much higher than reported here. Importantly, the processing, recovery, and resulting loss of Cd from islets did not occur in the renal cortex tissue that was measured for Cd and represented in Figure 6a. As such, there is a likelihood that the actual Cd content in islets is higher than that of the renal cortex in vivo. Additionally, if confirmed by other experiments, the recovery of islet function following Cd depletion suggests that Cd mediated inhibited islet insulin secretion represents a functional inhibition rather than a persistent toxicity.

The concentration of Cd found in the islets of 12 week Cd treated animals was roughly 7500 nmoles/gram protein (Figure 6a). This is a considerably higher Cd concentration previously reported by us in human islets with a median level of 26.1 nmoles/gram protein [36]. One explanation for this discrepancy between the two studies is that the source of human islets in the Wong 2017 report was the NIH/NIDDK sponsored Integrated Islet Distribution Program (IIDP). The processing, culture and shipment of these islets may have allowed for significant decreased Cd content if maintained in these conditions long term. Alternatively, species as well as type of exposure may account for the differences in islet Cd values. Certainly the route of administration by subcutaneous injection and relatively high level dose of Cd in the experimental animals is not reflective in typical human exposures. However, this accelerated dosing scheme is required to examine the long term biological effects of a metal that has a half-life measured in decades in a rodent with a life span a fraction of that time.

This study shows that islets isolated from nine-week Cd-treated animals had elevated basal (low glucose conditions) insulin release (Figure 3b) and significantly elevated insulin content (Figure 4b). By contrast, islets isolated from 12 week Cd-treated animals had lower glucose-stimulated insulin release (Figure 3c) and an almost statistically significant (2-way ANOVA; *p* = 0.07) decrease in insulin content (Figure 4c). This, taken with the increase in fasting blood glucose after 10 weeks of Cd treatment (Figure 1) would suggest that the animals may be insulin resistant at week 9 of Cd treatment and that pancreatic β-cell compensation is occurring as a response. However, not all of the results shown here support β-cell compensation. We did not find many significant changes in islet morphology and no changes in intra-islet insulin-positive β-cell numbers or cell density (Table 2 and Table 3). The β-cell compensation to insulin resistance is responsible for maintaining normal blood glucose levels and is characterized by increased β-cell mass, increased insulin biosynthesis, enhanced insulin release with increased sensitivity to glucose and other insulinotropic factors, for review see [37]. Eventually, β-cell failure occurs resulting in impaired glucose tolerance, eventual establishment of T2DM and the requirement for insulin drug therapy for patients [37]. In this study, β-cell failure is possibly occurring at week 12 of Cd treatment with decreased glucose stimulated insulin release (Figure 3c) and a lower, but not statistically significant, level of islet insulin content (Figure 4c). However, we did find a significant decrease in the number of islets per field of view or islet density when comparing low magnification H&E images from control and Cd-treated animals, see Figure 8. While the two-way ANOVA shows significant differences between treatment groups when the entire data set was examined, there were no differences detected between week-matched control- and Cd-treated animals. In addition, there was no apparent time-dependent effect with the greatest difference in control- vs. Cd-treatment mean values at the earliest six-week time point (Figure 8). Further work is needed to determine if Cd acts to diminish islet number following prolonged exposure.

The increase in islet zinc levels following six weeks vs. 12 weeks of Cd treatment may be of significance. Pancreatic β-cells have very high intracellular levels of Zn compared to any other cell type especially in secretory vesicles that contain insulin, for review see [38]. The diabetes risk genotype C/C at SNP rs13266634 of the SLC30A8 gene encoding the β-cell Zn transporter ZnT8 was associated with a higher total islet Zn concentration in human islets [36]. Presumably, the increase of Zn reported here was at least partly due to increased expression of metallthionein in response to Cd exposure as has previously been reported for other tissues as well as for in vitro exposed islets [22,39,40]. Interestingly, overexpression of metallothionein causes decreased glucose stimulated insulin secretion and decreased islet insulin content in C57BL/6J mice [41] as well as decreased pancreatic insulin content and increased islet metabolic dysfunction in non-obese diabetic mice [42]. If true, Cd may be indirectly causing impaired insulin secretion by way of inducing the expression of metallothionein. It is unclear what the effect the increase in Zn in response to Cd exposure will have on the physiology of islets and beta cells. It is also unclear on how the change in Zn concentration will affect Cd toxicity. Maintaining high Zn concentrations that are unique to beta cells is traditionally thought of as being essential to beta cell function. In addition to its traditional essential role in Zn associated proteins essential to any eukaryotic cell type, Zn is thought to play a unique role in beta cells. It has traditionally been assumed that Zn mediated crystallization of insulin in secretory vesicles contributes to the proper maturation, storage, and secretion of insulin. Additionally, paracrine functions were ascribed to Zn within pancreatic islets reviewed by [43]. However, the essentiality of maintaining the exceptionally high concentrations of Zn observed in beta cells has recently been called into question by studies that demonstrate that loss of function of the main Zn transporter responsible for maintaining the high concentration of Zn in islets and beta cells -ZnT8- is associated with a lower risk for diabetes [36,44].

Further studies for exploring potential additional mechanisms underlying the changes in Zn concentration such as changes in transporter expression are warranted. Exploring the location of each of Cd and Zn would be of high value in this regard. It is possible to use commercially available fluorprobes to detect intracellular cadmium. We recently reported on using divalent fluorophores to detect entry of Cd into MIN6 cells [45]. However, there are limitations as currently available Cd flurophores exhibit significant cross reactivity to other divalent metals. Other divalent cations such as Zn and Ca alter the fluorescence and data generated by these probes, rendering them of limited use for the accurately studying interactions between the two elements. A member of this collaboration—the Muayed lab—has published on imaging trace elements in Cd exposed cells -the insulin secreting beta cell line MIN6 [46]. However, this method is still in need of further refinement in order to assist in providing insights on whether or not Cd and Zn co-localize in beta cells.

While the importance of β-cell failure in the context of the development of T2DM is undetermined and an active area of research, it is known that β-cells are some of the most metabolically active with cell function being especially sensitive to reactive oxygen species (ROS), for review see [47]. Importantly, some consider alterations in β-cell function and not necessarily loss of β-cell number sufficient for the progression to T2DM [37]. Future studies are needed to elucidate the mechanism(s) by which Cd may cause ROS formation in β-cells and subsequent changes in β-cell signaling and function.

## 4. Materials and Methods

### 4.1. Animals

Male Sprague-Dawley rats weighing 240–300 g received daily subcutaneous injections of Cd (0.6mg (5.36 µmoles)/kg in 0.25–0.40 mL of isotonic saline, five days per week, for up to 12 weeks) in the form of CdCl_2_, while control animals received the saline alone. Weekly, 24 h fasting blood samples were collected from control and Cd-treated animals. All animals were purchased from Envigo RMS (Indianapolis, IN, USA). All animal studies were approved by the Institutional Animal Care and Use Committee of Midwestern University and carried out in an AAALAC accredited animal facility; IACUC file # 1963, original approval date 4/2013.

### 4.2. Islet Isolation and Treatment

Animals were anesthetized with ketamine/xylazine (67/7 mg/kg; i.p.) then the abdominal cavity opened, pancreas removed and islets isolated using a modified procedure from [48]. Pancreatic tissue was placed in ice-cold sterile-filtered, Hanks Buffered Salt Solution (HBSS) (CaCl_2_: 0.14 g/L, MgCl_2_–6H_2_O: 0.1 g/L, MgSO_4_: 0.049 g/L, KCl: 0.4 g/L, KH_2_PO_4_: 0.06 g/L, NaCl: 8g/L, Na_2_HPO_4_: 0.048 g/L, D-glucose: 0.05g/L) then thoroughly minced with scalpel blades. Floating pieces of fat and mesenteric tissue were discarded. The minced pancreatic tissue was then placed into a screw top container with approximately 85 mg (19,000 units) of type IV collagenase (Worthington Biochemical Corp., Lakewood, NJ, USA, cat. #LS004188) dissolved in 5 mL of HBSS. The tissue/enzyme mixture was placed in a water bath at 37 °C and stirred using a magnetic rod for 12 to 16 min or until no visible tissue chunks remained. Next, 10 mL of ice cold HBSS was added to stop collagenase activity. After one minute the liquid supernatant was removed and discarded. Fifteen mL of room temperature HBSS was added to the remaining sediment and then allowed to settle for 30 s. The supernatant was removed and the sediment resuspended eight more times with 15 mL of HBSS each time, the last four resuspensions using ice cold HBSS. After removing the supernatant from the last resuspension, the remaining sediment, which consists of islets and small clumps of undigested acinar cells, was transferred into a petri dish and viewed under a low power inverted microscope. Individual islets were identified and transferred using a 1 mL pipette into six-well cell culture dish. One islet was placed in a single well containing 1 mL

RPMI (Invitrogen, Carlsbad, CA, USA cat. #61870) with 10% heat inactivated FBS (Invitrogen, cat. #16140) and penicillin/streptomycin (Invitrogen, cat. #15140-122). Isolated islets were allowed to recover from the isolation procedure for 90 min in a humidified chamber at 37 °C and 5% CO_2_. Then individual islets were rinsed twice with serum-free HBSS and incubated in HBSS containing low/basal (0.5 mg/mL) or high (3.0 mg/mL) concentrations of glucose for 4 h.

After islets were incubated for 4 h in low or high glucose conditions, aliquots of HBSS were taken and stored at −80 °C for later insulin determination. Then islets were rinsed in normal glucose HBSS and lysed with 0.5 mL of a commercially available lysing buffer, Mammalian Protein Extraction Reagent (MPER; Thermo Scientific, Waltham, MA, USA cat. #78501). Aliquots of the lysing buffer were stored at −80 °C until insulin, Cd or Zn content could be determined.

### 4.3. Measurement of Insulin from Serum and Isolated Islets

Serum samples obtained from 5 h fasted animals were used to determine insulin levels in 6, 9, and 12 week treated animals. Blood samples were taken from the exposed vena cava immediately before euthanasia while under ketamine/xylazine anesthesia. Blood was allowed to clot at room temperature for 30 min then were centrifuged (2000× *g*) for 15 min at 4 °C. Resulting clear serum separated from the remaining clotted blood and stored at −80 °C for further analysis. Insulin levels were quantified using a commercially available ELISA kit (Crystal Chem Inc., Downers Grove, IL, USA, cat. #90060).

### 4.4. Measurement of Cd and Zn Content in Freshly Isolated Pancreatic Islets, Whole Pancreata and Renal Cortex

For metal analysis, dried tissue samples were hydrolyzed in trace metal grade 70% NHO_3_ (Optima grade, Fisher Scientific, Pittsburgh, PA, USA) at 70 °C. Protein concentrations were measured in a subsample using the micro BCA kit following dilution and pH neutralization with trace metal grade NaOH (Fisher Scientific). Cd and Zn concentrations were measured by inductively coupled plasma mass spectrometry (ICP-MS) as previously described in [22]. Data shown in Figure 5 were measured by Chemical Solutions, Inc. (Mechanicsburg, PA, USA). Cd and zinc metal content in Figure 6a,b were measured at the Northwestern University Quantitative Bioelemental Imaging Center.

### 4.5. Insulin and Glucagon Immunolabelling of Pancreatic Islets

Pancreata were sectioned at 5 µm thickness then deparaffinized and rehydrated in a series of washes of decreasing concentrations of ethanol. The slides were rinsed under cold tap water. Antigen retrieval consisted of boiling slides in Tris-EDTA buffer (1.21 g Tris, 0.37 g EDTA, 1000 mL distilled water, and 10 mL Triton X-100), pH 9.0 for 30 min and then rinsed again under tap water. The slides were then washed in 0.1 M Phosphate Buffered Saline (PBS; Gibco, Waltham, MA, USA cat. #70011). After washing, the slides were blocked with 1% goat serum (Sigma, St. Louis, MO, USAcat. #G6767) solution for one hour. After blocking, primary antibody diluted in 1% goat serum was applied incubated overnight at 4 °C. The next day, after rinsing unbound primary antibody in 0.1 M PBS, secondary antibody diluted in 1% goat serum was applied and incubated for 2 h. After secondary incubation, the slides were rinsed in 0.1 M PBS. Then mounting media and coverslips were added, left to dry overnight, and examined within the next two days. The following primary and secondary antibodies were used: rabbit polyclonal insulin antibody (1:200, Santa Cruz Biotechnology, Dallas, TX, USA cat. #9168), mouse monoclonal glucagon antibody (1:50, Santa Cruz Biotechnology, cat. #57171), goat anti-rabbit fluorescein (1:50, Thermo Scientific, cat. #31635) and goat anti-mouse rhodamine (1:100, Thermo Scientific, cat. #31660). For each tissue section, approximately nine images of individual islets were captured from each animal with each islet considered an *n* value of one; images from at least four different animals from each treatment at each time point were used.

### 4.6. Islet Morphometric Analyses

For morphometric analyses, pancreata were sectioned (5 µm thickness) and stained with hematoxylin and eosin (H&E) at AML Laboratories (St. Augustine, FL, USA). Tissue samples were examined with a Nikon Eclipse E400 microscope and digital images were taken using an Evolution MP Color digital camera (Media Cybernetics, Rockville, MD, USA). Image-Pro Plus software (Media Cybernetics, Rockville, MD, USA) was used to capture and quantify islet morphology. For each tissue section, approximately nine images of individual islets were captured from each animal with each islet considered an *n* value of one; images from at least four different animals from each treatment at each time point were used.

### 4.7. Histopathological Analyses of Islets of Langerhans

H&E stained sections of pancreata (5 µm thick) were assessed by a trained veterinarian pathologist (Colorado HistoPrep, Fort Collins, CO, USA). For semi-quantitative scores: 0 = no significant findings, 1 = minimal, 2 = mild, 3 = moderate, 4 = moderately severe and 5 = severe.

### 4.8. Quantification of Islet Density in Pancreatic Tissue

H&E stained sections of pancreata (5 µm thick) were used to assess islet density (number of islets per field of view) from images captured using a 10× objective. Two to three fields of view were captured per tissue section from an individual animal and averaged for an *n* value of 1; *n* ≥ 3 for each treatment group.

### 4.9. Statistical Analyses

Analyses were performed using the GraphPad Prism, San Diego, CA, USA (v. 9.0.0). Data were evaluated by Student’s T-test, or if appropriate, two-way or one-way ANOVA followed by post-hoc Tukey’s test; *p* ≤ 0.05 was considered significantly different. All numerical data are mean ± standard error of the mean (SEM).

## 5. Conclusions

Overall, this work provides insights into the diabetogenic effects of the ubiquitous environmental pollutant, Cd, and challenges our accepted views of Cd accumulation and redistribution in vivo. Specifically, this work highlights the islets of Langerhans as potential targets of Cd accumulation and toxicity with altered β-cell function as one likely mechanism of the diabetogenic effects of Cd. Additionally, the rapid efflux of Cd following glucose stimulated insulin secretion shown here has implications on the half-life of Cd in islets of Langerhans which may be longer in other tissues such as the renal proximal tubules. We also observed an increase in islet Zn concentration in response to Cd accumulation. It is likely that upregulation of metallothionein—the main biological divalent metal buffer [22] contributed to this observation. This increase in metallothionein could then adversely affect β-cell function [41,42] and be an indirect mechanism by which Cd alters insulin secretion. However, additional mechanisms warrant exploration. Future studies need to focus on the likely mechanisms by which Cd causes acute β-cell dysfunction (e.g., role of L-type voltage gated calcium channels), accumulation and transport (i.e., ZnT8 zinc transporter), as well as long term cell function (ROS generation and islet density and size).

## Figures and Tables

**Table 1:** Hematoxylin and eosin (H&E) stained pancreatic islets from cadmium- and saline-treated animals were evaluated by a trained veterinary pathologist for: apoptosis, pyknosis, vacuolation and dissociation.

	Apoptosis	Pyknosis	Vacuolation	Dissociation
Control Week 9	0 ± 0	1 ± 0	1.7 ± 0.3	0 ± 0
Cadmium Week 9	0.8 ± 0.6	1.7 ± 0.7	2.3 ± 0.3	1.2 ± 0.4
Control Week 12	0 ± 0	1 ± 0	2.3 ± 0.3	0 ± 0
Cadmium Week 12	0.7 ± 0.3	2 ± 0	2.6 ± 0.3	0.8 ± 0.2

For semi-quantitative scores: 0 = no significant findings, 1 = minimal, 2 = mild, 3 = moderate, 4 = moderately severe and 5 = severe. Data are mean ± SE, *n* = 3 for all treatment groups. No statistically significant differences were detected between week-matched control and Cd-treated animals; Mann–Whitney *p* > 0.05.

**Table 2:** All values are mean ± SEM; ranges are also given underneath actual values. Data show changes in pancreatic islet morphology in animals exposed to Cd (0.6 mg/kg/day) for up to 12 weeks and saline-control animals. Pancreata from a minimum of four animals from each treatment group and time point were examined.

Characteristics	Week 6		Week 9		Week 12		
	Control	Cd	Control	Cd	Control	Cd	*p*-Value
Cells/Surface Area	* 0.12 ± 0.001	0.09 ± 0.001	* 0.125 ± 0.001	0.1 ± 0.001	* 0.12 ± 0.001	0.11 ± 0.001	0.04
Dia Mean (µm)	118 ± 9 (38–236)	108 ± 20 (39–219)	77 ± 7 (36–249)	123 ± 9 (46–243)	134 ± 9 (41–289)	127 ± 9 (40–282)	0.062
Dia Min (µm)	100 ± 4 (32–254)	88 ± 17 (29–135)	* 63 ± 6 (12–186)	102 ± 7 (33–243)	111 ± 8 (43–245)	105 ± 7 (29–256)	0.046
Dia Max (µm)	141 ± 11 (43–327)	131 ± 25 (49–269)	93 ± 9 (43–311)	130 ± 11 (41–369)	165 ± 11 (42–366)	157 ± 12 (46–368)	0.194
Roundness	1.20 ± 0.014 (1.05–1.55)	1.12 ± 0.031 (1.12–1.45)	1.21 ± 0.016 (1.09–1.54)	1.19 ± 0.019 (1.08–1.66)	1.22 ± 0.017 (1.08–1.65)	1.23 ± 0.025 (0.06–1.64)	1.000
Surface Area (µm^2^)	13,918 ± 2057 (1172–16,2802)	14,256 ± 4012 (1270–38,503)	6209 ± 1484 (993–49,836)	15,112 ± 2197 (1111–35,201)	18,245 ± 2234 (1105–66,452)	16,544 ± 2296 (1470–58,057)	0.151
Perimeter (µm)	205 ± 16 (65–491)	192 ± 36 (72–382)	135 ± 14 (61–471)	216 ± 18 (79–569)	237 ± 17 (62–489)	224 ± 16 (74–530)	0.051
Number of Nuclei	86 ± 12 (5–338)	68 ± 20 (6–178)	43 ± 10 (4–348)	81 ± 11 (7–273)	115 ± 14 (10–379)	96 ± 13 (6–382)	0.136

An asterisk (*) indicates statistically significant differences compared to week-matched Cd-treatment group (two-way ANOVA followed by Tukey’s multiple comparison test, *p* ≤ 0.05); *n* ≥ 9 per treatment group per time point.

**Table 3:** All values are mean ± SEM; ranges are also given underneath actual values. Pancreata from a minimum of four animals from each treatment group and time point were examined.

Characteristics	Week 9		Week 12		
	Control	Cd	Control	Cd	*p*-Value
Insulin & Glucagon Ratio	3.15 ± 0.81 (1.33–6.0)	9.08 ± 3.1 (2.95–17)	4.02 ± 1.6 (2.04–10)	3.32 ± 0.51 (2.75–5.8)	0.088
Insulin/SA (#/µm2)	3.81 × 10^−3^ ± 4.1 × 10^−4^ (2.70 × 10^−3^–5.22 × 10^−3^)	4.12 × 10^−3^ ± 3.24 × 10^−5^ (3.20 × 10^−3^–4.61 × 10^−3^)	4.18 × 10^−3^ ± 3.80 × 10^−4^ (3.4 × 10^−3^–5.53 × 10^−3^)	3.42 × 10^−3^ ± 6.63 × 10^−5^ (1.97 × 10^−3^–5.54 × 10^−3^)	0.266
Glucagon/SA (#/µm^2^)	1.63 × 10^−3^ ± 6.00 × 10^−4^ (6.97× 10^−5^–3.04 × 10^−3^)	7.00 × 10^−4^ ± 2.00 × 10^−4^ (9.91× 10^−5^–7.94 × 10^−3^)	1.41 × 10^−3^ ± 2.00 × 10^−4^ (5.53 × 10^−5^–1.94 × 10^−3^)	1.14 × 10^−3^ ± 3.24 × 10^−4^ (7.96 × 10^−5^–2.47 × 10^−3^)	0.380
# of Gluc Nuc/5000 µm (#/µm)	6 ± 2 (3–14)	3 ± 1 (1–4)	5 ± 1 (2–7)	4 ± 1 (2–9)	0.388
# of Ins Nuc/5000 µm(#/µm)	14 ± 2 (10–19)	15 ± 1 (12–19)	15 ± 1 (13–20)	13 ± 3 (7–20)	0.273

Data were analyzed using two-way ANOVA; *n* ≥ 9 per treatment group per time point and expressed as number per surface area (#/µm^2^).

## Data Availability

All data presented in this study are readily available upon request from the corresponding author.

## Abbreviations

T2DM	Type-2 diabetes mellitus
ICP-MS	Inductively-coupled plasma mass spectrometry
Cd	Cadmium
Zn	Zinc
